# Observation of optic disc neovascularization using OCT angiography in proliferative diabetic retinopathy after intravitreal conbercept injections

**DOI:** 10.1038/s41598-018-22363-0

**Published:** 2018-03-05

**Authors:** Xiao Zhang, Chan Wu, Li-jia Zhou, Rong-ping Dai

**Affiliations:** 1 Department of Ophthalmology, Peking Union Medical College Hospital, Peking Union Medical College, Chinese Academy of Medical Sciences, Beijing, China; 2 Department of Ophthalmology, Beijing Hepingli Hospital, Beijing, China

## Abstract

This study reports the short-term efficacy and safety of intravitreal conbercept injections for neovascularization at the disc (NVD) in patients with proliferative diabetic retinopathy (PDR). Conbercept is a recombinant fusion protein with a high affinity for all isoforms of vascular endothelial growth factor (VEGF)-A, placental growth factor and VEGF-B. A prospective case series study was conducted in 15 patients (15 eyes). Patients had complete ocular examinations and received a 0.5 mg intravitreal conbercept injection followed by supplemental pan-retinal photocoagulation (PRP). Optical coherence tomography angiography (OCTA) was performed before and after treatment. Before treatment, the mean NVD area was 1.05 ± 0.33 mm^2^, and it decreased to 0.56 ± 0.17 mm^2^ after an interval of 7.5 d (p = 0.000). One eye required vitrectomy during follow-up. Recurrent NVD was observed in 2 eyes, which resolved after repeated injections. The remaining 12 eyes were stable over a mean follow-up period of 8.3 months. The mean area of the NVD in 14 patients without vitrectomy was 0.22 ± 0.11 mm^2^ (p = 0.000) at the last visit. Intravitreal conbercept injections combined with intensive PRP are an effective and safe treatment for PDR with NVD. Quantitative information on NVD can be obtained with OCTA, which may be clinically useful in evaluating the therapeutic effect.

## Introduction

Diabetes mellitus (DM) is one of the world’s fastest growing chronic diseases, and diabetic retinopathy (DR) is a specific microvascular complication of DM. As the prevalence of DM continues to increase, DR is still the leading cause of acquired vision loss worldwide in middle-aged and therefore economically active people^1^. Proliferative diabetic retinopathy (PDR) is a severe stage of DR and one form of vision-threatening DR. Pan-retinal photocoagulation (PRP) is the established treatment for PDR, as it is effective and has preserved vision in numerous patients over the past several decades^2–4^.

As vascular endothelial growth factor (VEGF) plays a role in the process of neovascularization in PDR, anti-VEGF agents including ranibizumab and bevacizumab have been assessed for the treatment of PDR in recent years, and the results were satisfactory^5–9^. Conbercept is a recombinant fusion protein that consists of the VEGF binding domains of the human VEGFR-1 and VEGFR-2 combined with the Fc portion of the human immunoglobulin G1. In addition to having a high affinity for all isoforms of VEGF-A, it also binds to placental growth factor (PlGF) and VEGF-B^10^. As the use of conbercept in the treatment of PDR is off-label, there are few reports concerning this topic. We have conducted a prospective study on the short-term efficacy and safety of intravitreal conbercept injections combined with laser treatment for neovascularization at the disc (NVD) in patients with PDR.

The introduction of optical coherence tomography angiography (OCTA) into clinical practice allows the detection and visualization of the blood flow and morphology of retinal vessels and is useful to monitor different NVD subtypes, their development, and the efficacy of treatment regimens as well as to define new vessel morphological details^11,12^. To obtain quantitative information on NVD, OCTA was performed before and after treatment, and the NVD area was calculated.

## Methods

### Patients

This prospective case series included 15 eyes from 15 patients with diabetes who presented at the Peking Union Medical College Hospital between January and September 2016. They had been clinically diagnosed with PDR with active NVD. All the patients were informed about the purpose and treatment procedure, and written informed consent were obtained before intravitreal injections. All procedures conformed to the tenets of the Declaration of Helsinki. The methods were carried out in accordance with the approved guidelines, and the study was approved by the Ethics Committee of the Peking Union Medical College Hospital.

Inclusion criteria included patients with PDR with disc neovascularization, patients with clear media that allowed fundus examinations and OCT angiography examinations, and patients who had not been treated with intravitreal anti-VEGF or steroid injections within six months. Exclusion criteria included patients with opaque media that influenced fundus examination, including corneal opacity, cataracts and vitreous hemorrhages; previous eye diseases such as glaucoma and retinal diseases besides DR, such as retinal vein occlusion or AMD; eye infections and intraocular inflammations; tractional retinal detachment; and penetrating ocular trauma or intraocular surgery within the previous 6 months prior to intravitreal injection. Patients with uncontrolled hypertension, recent myocardial infarction or cerebral vascular incidents were also excluded.

### Ocular examination

All patients had a complete ocular examination before and after treatment that included the following measurements: best corrected visual acuity (BCVA) using the International Standard Visual Acuity Chart, intraocular pressure (IOP) using a non-contact tonometer, ocular motility, pupillary light reflex, slit-lamp examination, fundus examination using indirect ophthalmoscope, fundus photography (TOPCON TRC-NW6S Non-Mydriatic Retinal Camera), spectral domain optic coherence tomography, and OCT angiography (OptoVue, Freemont, California).

AngioVue disc examinations were performed by one experienced eye specialist (ZLJ). The scanning pattern was 4.5 × 4.5 mm^2^. As OCT angiography produces multiple scans, the best image for the detection of new vessel architecture was selected for use in the study. The B-scan OCT was used to confirm the presence of NVD, and new vessels were observed as preretinal proliferative structures or protrusions into the vitreous cavity with positive flow signals. Segmentation of the inner border of the B-scan was manually moved to the vitreous cavity, just above the new vessels, and the outer border was adjusted to minimize the depiction of the superficial vascular plexus^13^.

### Quantitative evaluation of NVD

A circle with a 3-mm diameter was drawn with its center at the optic disc, and the structure of the NVD was outlined with white lines using Adobe Photoshop (CS6), with the background colored black. The processed image was saved as a black and white two-color chart. If ${{\rm{N}}}_{{NVD}}$ and ${{\rm{N}}}_{0}$ are the numbers of pixels of the NVD (white lines) and the circle, respectively, then the area of the NVD can be calculated as.

$${\rm{A}}={\rm{\pi }}{{\rm{d}}}^{2}\frac{{{\rm{N}}}_{{NVD}}}{4{{\rm{N}}}_{0}}$$

**Figure 1:**
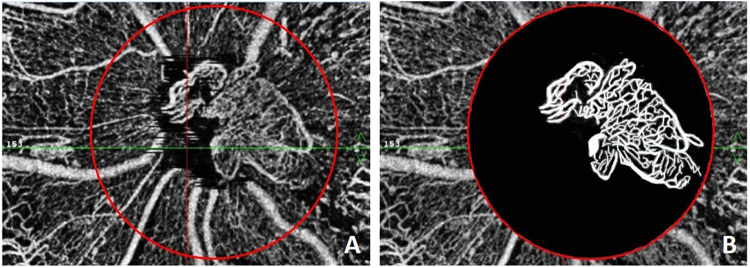
The procedure to measure the neovascularization at the disc (NVD). (**A**) A circle with a 3-mm diameter was drawn that was centered at the optic disc; (**B**) The structure of the NVD was outlined with white lines, and the background was colored black.

Measurements of the NVD area represent the average value calculated by two eye specialists (DRP, ZX) who were blinded to the image origins. The measurement procedure is shown in Fig. 1.

### Intravitreal injections

All patients received an explanation of the off-label use of conbercept in PDR and its potential benefits and risks. Informed consents were obtained, and a prophylactic topical antibiotic (Levofloxacin) was instilled for 3 days before treatment.

After topical anesthesia and sterilization of the operating field, 0.5 mg/0.05 mL of conbercept was injected intravitreally at the inferotemporal pars plana (4 mm posterior to the limbus). After the injection, the intraocular pressure was measured, and prophylactic topical antibiotic drops were instilled for 3 days. Intensive retinal laser photocoagulation was applied beginning two weeks after the intravitreal injection. A slit-lamp laser was used, and patients received laser treatment under topical anesthesia. For patients without previous PRP, laser therapy was completed after three to four sessions, with a total of 1600 to 1800 burns. For patients with previous PRP, laser treatment was applied where the former laser treatment was not sufficient.

### Statistical analysis

SPSS statistical software version 20.0 (SPSS Inc, Chicago, Illinois) was used for statistical analysis. A paired-samples t-test was used to compare the area of the NVD before treatment and after intravitreal conbercept injection. A probability (P) value < 0.05 was considered statistically significant.

## Results

A total of 15 patients (15 eyes) with type 2 diabetes mellitus were investigated in this study. Of the 15 patients, 7 were male and 8 were female. The mean age was 42.9 ± 10.9 years (range 29–64 years), and the demographic details are shown in Table 1. Ten eyes had undergone previous PRP treatment and presented with obvious NVD, manifested as repeated vitreous hemorrhages or obvious leaking of the NVD seen during FA examination. Five eyes were diagnosed as high-risk PDR without previous PRP treatment, manifested as NVD.

**Table 1:** Demographic and OCTA^a^ features of the studied eyes.

No.	Initials	Gender	Age	Eye	Before treatment	First visit after IVC^b^		Last visit after IVC and PRP or supplement PRP^d^	
					Area of NVD^c^ (mm^2^)	Period (d)	Area of NVD (mm^2^)	Follow-up time (m)	Area of NVD (mm^2^)
1	LF	F	42	R	1.08	7	0.55	6	0.34
2	DXY	M	37	L	0.97	9	0.25	Vitrectomy	—
3	LDX	M	36	R	1.06	7	0.69	12	0.25
4	LKM	F	64	L	1.27	7	0.83	14	0.07
5	ZXJ	M	51	L	1.03	7	0.62	6	0.27
6	WSX	F	62	R	1.13	7	0.71	14	0.24
7	YM	F	30	R	0.89	9	0.53	6	0.20
8	SQ	M	38	R	1.57	7	0.71	13	0.50
9	YZF	M	50	L	1.27	7	0.65	6	0.22
10	LS	M	33	R	1.55	7	0.48	6	0.25
11	XAH	F	52	L	0.53	9	0.40	8	0.19
12	LHH	F	46	R	0.34	6	0.25	6	0.09
13	LJZ	M	29	R	1.18	7	0.64	7	0.20
14	CJ	F	35	L	0.96	9	0.60	6	0.18
15	SWY	F	38	R	0.89	7	0.49	6	0.14

^a^Optical coherence tomography angiography.

^b^Intravitreal conbercept.

^c^Neovascularization at the disc.

^d^Pan-retinal photocoagulation.

In fundus biomicroscopy or color photography, new vessels of the optic disc usually manifest as irregular blood vessels on the optic nerve head or protruding into the vitreous cavity; however, the detailed structures of the NVD are sometimes not clear and simply manifest as redness of the optic papilla or an unclear optic disc border. During FA examination, the neovascularization of the disc is clear for only a few seconds, and then the flowing dye leakage makes it difficult to visualize the vessel profile. During OCT angiography, we were able to observe the detailed structures of the NVD clearly. In the 15 eyes, most of the NVD manifested as an irregular proliferation of fine vessels on or just near the optic disc (Fig. 1 Left).

**Figure 2:**
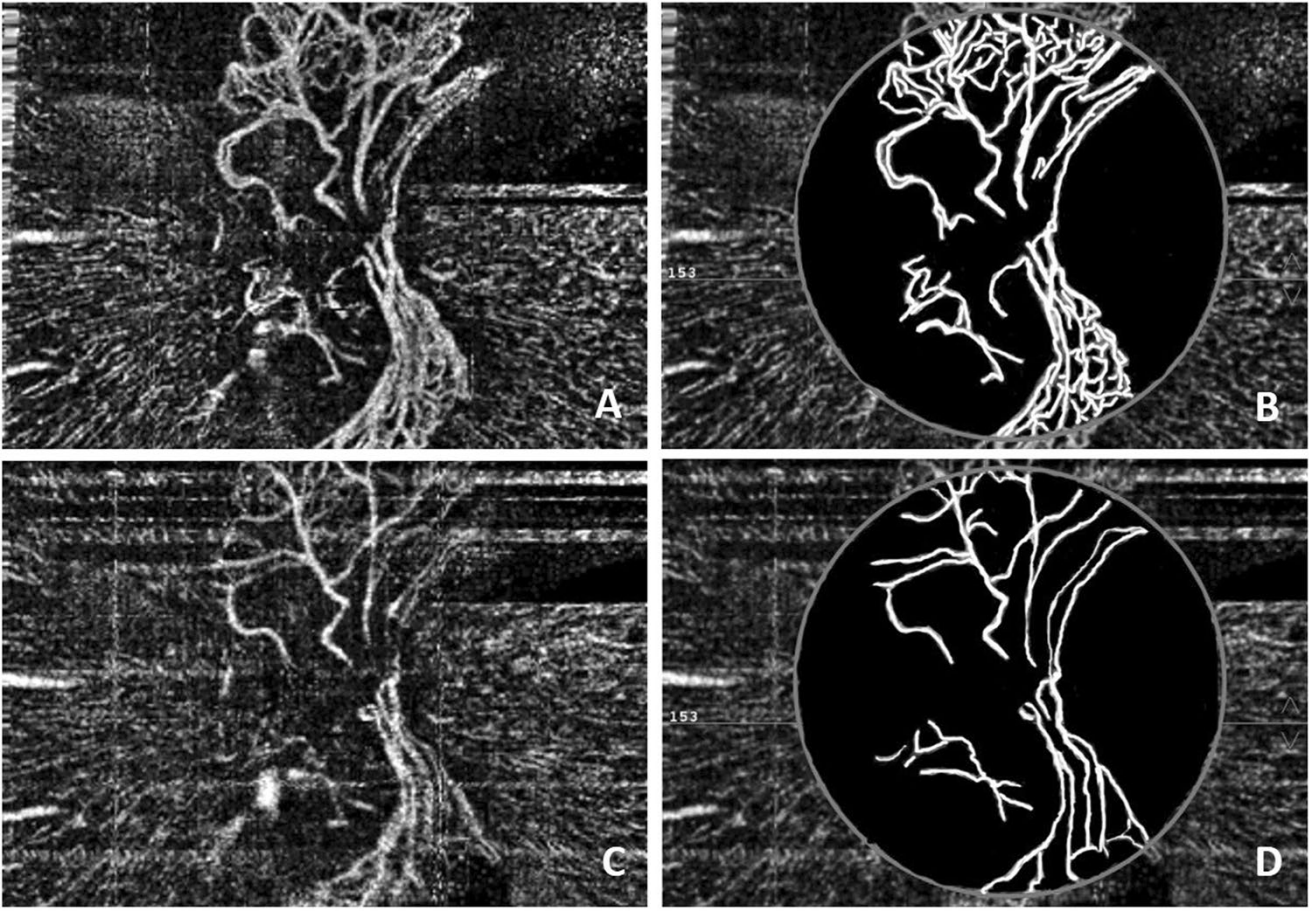
Male, 50 years old, NVD of the left eye. (**A**) OCTA showed obvious NVD before treatment; (**B**) The processed image, where the area of the NVD was 1.27 mm^2^. (**C**) OCTA showed a significant decrease of the NVD 7 days after intravitreal conbercept therapy; (**D**) The processed image, where the area of the NVD was 0.65 mm^2^.

The changes in the NVD after intravitreal conbercept therapy combined with laser treatment were quantitatively observed. Before treatment, the mean area of the NVD was 1.05 ± 0.33 mm^2^ (range 0.34–1.57 mm^2^). The time to the first visit after the initial intravitreal conbercept therapy ranged from 6 to 9 days (mean 7.5 days). The mean area of the NVD decreased to 0.56 ± 0.17 mm^2^ (range 0.25–0.83 mm^2^) (Table 1). The area of the NVD decreased significantly after intravitreal conbercept (t = 7.426, p = 0.000) (Fig. 2). Twelve eyes that were stable underwent laser treatment after the intravitreal injection. There were no enlarged areas of prior NVD or new NVD noted in these eyes during the follow-up period. One case with an ocular complication showed a vitreous hemorrhage and proliferative membrane formation after the intravitreal injection and laser treatment, and this patient (1/15, 6.7%) underwent vitrectomy surgery. The NVD in another two eyes had regrown 10 weeks and 12 weeks after the injection, manifesting as an enlarged NVD area with a minor vitreous hemorrhage in one eye. These two patients were treated with repeated injections of conbercept and enhanced laser treatment. The NVD regressed in both eyes, and the vitreous hemorrhage was resolved at the last visit.

**Figure 3:**
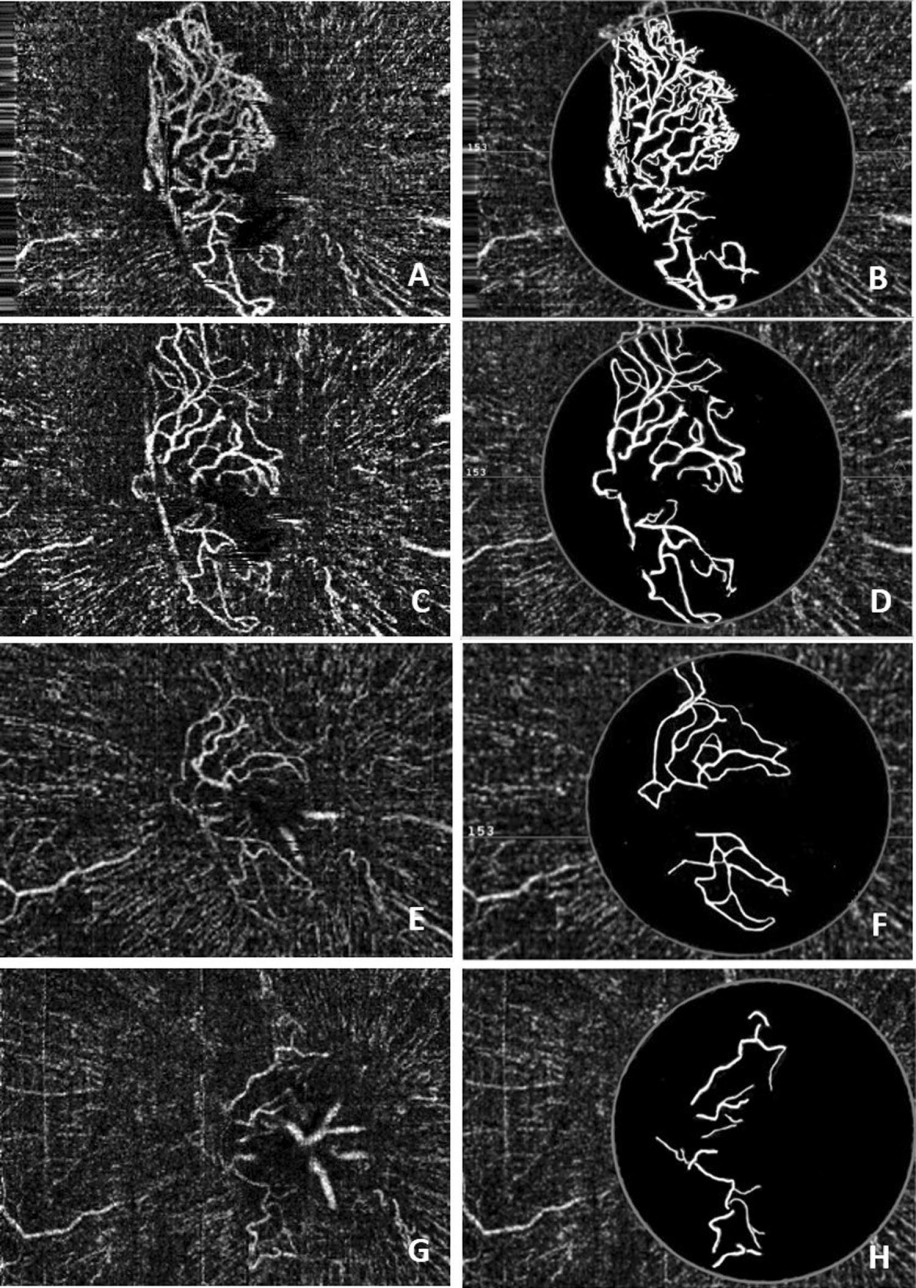
Female, 62 years old, NVD of the right eye. (**A**) Before treatment, OCTA showed obvious NVD and exuberant vascular proliferation (EVPs) in the upper margin; (**B**) The processed image, where the area of the NVD was 1.13 mm^2^. (**C**) One week after intravitreal conbercept therapy, OCTA showed an alleviation of the NVD and the disappearance of EVPs; (**D**) The processed image, where the area of the NVD was 0.71 mm^2^. (**E**) OCTA image of the NVD one month after intravitreal conbercept therapy and laser treatment; (**F**) The processed image, where the area of the NVD was 0.39 mm^2^. (**G**) OCTA image of the NVD 14 months after intravitreal conbercept therapy and laser treatment; (**H**) The processed image, where the area of the NVD was 0.24 mm^2^.

**Figure 4:**
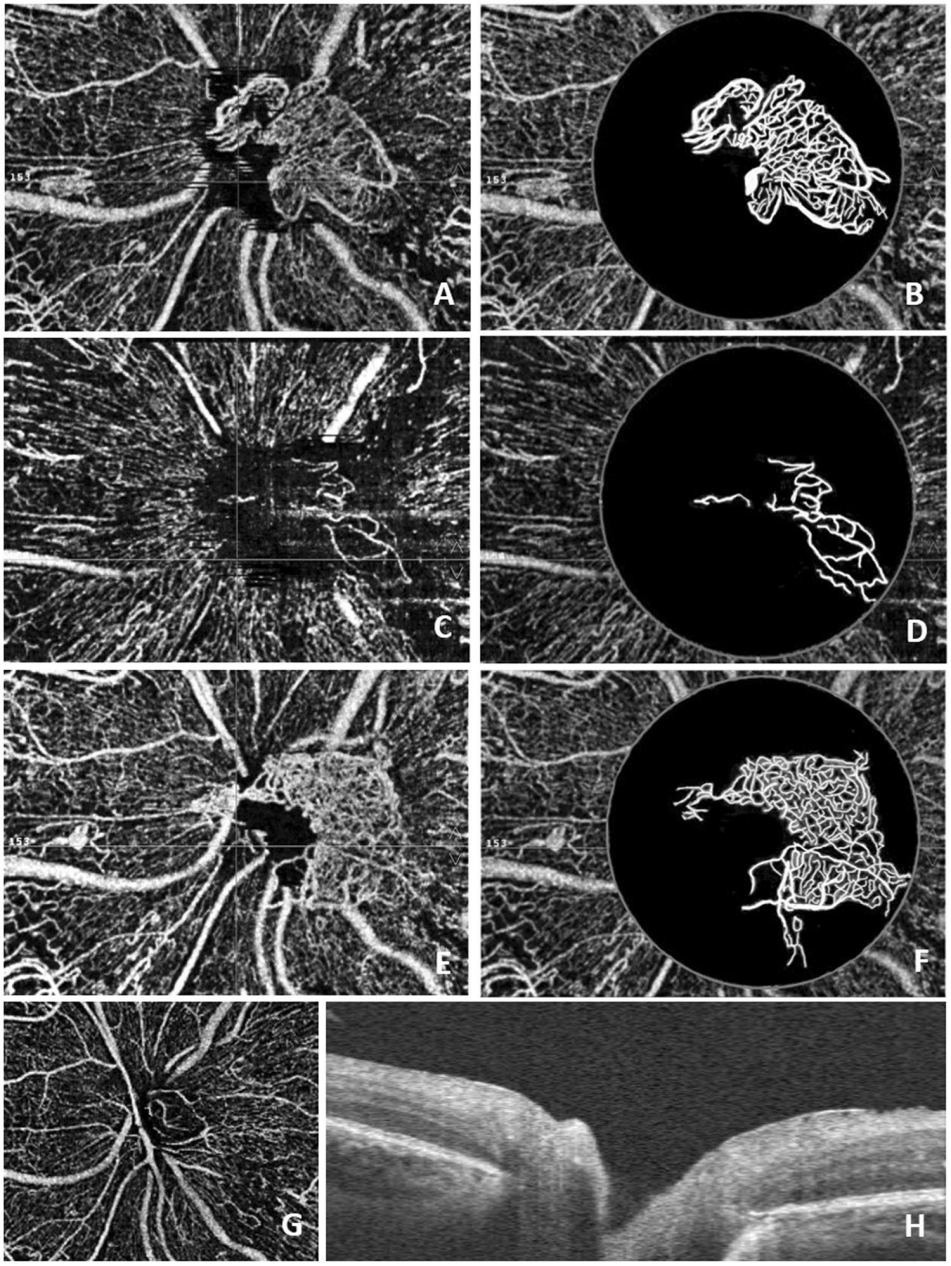
Male, 37 years old, NVD of the left eye. (**A**) Before treatment, OCTA showed obvious NVD and EVPs; (**B**) The processed image, where the area of the NVD was 0.97 mm^2^. (**C**) Nine days after intravitreal conbercept therapy, OCTA showed an alleviation of the NVD and the disappearance of EVPs; (**D**) The processed image, where the area of the NVD was 0.25 mm^2^. (**E**) OCTA showed NVD regrowth and EVPs reappearance 8 weeks after intravitreal conbercept therapy and laser treatment; (**F**) The processed image, where the area of the NVD was 1.01 mm^2^. (**G**) The patient underwent vitrectomy surgery because of a vitreous hemorrhage and proliferative membrane formation. Four months after surgery, OCTA showed a normal structure of the optic nerve head without NVD; (**H**) B-scan OCT confirmed the absence of NVD.

The mean follow-up period was 8.3 months (range: 6–14 months). The mean area of the NVD in 14 patients without vitrectomy was 0.22 ± 0.11 mm^2^ (range 0.07–0.50 mm^2^) at the last visit (Table 1). Compared with the data before treatment, the area of the NVD decreased significantly after treatment (t = 10.627, p = 0.000) (Fig. 3). The NVD disappeared after vitrectomy in 1 patient (Fig. 4). The BCVA on the International Standard Visual Acuity Chart ranged from FC to 0.6 at baseline. Three of the 15 eyes showed an improvement in BCVA of more than 3 lines, 12 eyes showed stable BCVA, and none had loss of vision greater than one line at the last visit. No systemic adverse events were observed following conbercept injections.

## Discussion

Diabetic retinopathy remains a major cause of visual impairment and blindness in the world. Over one-third of the world’s 285 million people with DM are estimated to have DR, and one third of these (approximately 31.7 million) have vision-threatening diabetic retinopathy (VTDR)^14^. According to the World Health Organization (WHO), it is estimated that DR accounts for 4.8% of the cases of blindness (37 million) worldwide^15^.

Laser photocoagulation is the traditional treatment for PDR, and PRP has been effective at preserving vision in numerous PDR patients over the past several decades. According to the Diabetic Retinopathy Study, PRP reduced the risk of severe visual loss by more than 50%^2–4^. However, this is not always the case, as approximately 60% of patients with PDR who respond to scatter laser treatment show regression of neovascularization within 3 months, and many require additional laser treatments^16,17^. Ten out of 15 eyes in our study had previous PRP treatment, but NVD was still active, and further treatment was needed. Neovascularization on and around the optic disc and vitreous hemorrhages were found to be more frequently associated with severe visual loss despite PRP in the Diabetic Retinopathy Study (DRS) and ETDRS^7^. Meanwhile, PRP is impossible to apply completely in patients with media opacities such as vitreous hemorrhages or cataracts. It has been reported that despite frequent follow-up examinations and timely PRP, the 5-year cumulative rate of pars plana vitrectomy in ETDRS patients is 5.3%^17^. Therefore, the need exists for an adjunctive therapy for PDR.

As VEGF plays a role in the process of neovascularization in many ocular vascular diseases, anti-VEGF agents have been frequently used for the treatment of PDR in recent years. Bevacizumab was the first anti-VEGF agent used for PDR. It is reported to be a well-tolerated medication that causes regression of abnormal diabetic neovascularizations and a rapid resolution of vitreous hemorrhages^5–8^. Avery *et al*. studied the angiographic leakage from abnormal new vessels after intravitreal bevacizumab injections and showed that all patients with neovascularizations seen with fluorescein angiography (44 eyes) had an at least partial or complete reduction of leakage from the neovascularization within 1 week after the injection^5^. Minnella *et al*. also reported regression and reduction of the area of new vessels as early as 1 month after intravitreal injection in all studied eyes and a clearing of the bleeding in eyes with recurrent vitreous hemorrhages. These effects were maintained for 3 months in all eyes and tended to be stable at 9 months with no side effects from the injections^18^. In the series reported by Ababneh *et al*., more than 50% of the new vessels were stable or improved at 6 months^6^. Arevalo *et al*.^19^ and Arevalo and Garcia-Amaris^7^ found that 27 eyes (61.4%) with retinal neovascularization completely regressed after intravitreal bevacizumab injections, and 15 eyes (34.1%) had a partial regression of retinal neovascularization seen via fundus examination and fluorescein angiography, whereas 2 eyes of 2 patients (4.5%) had no regression of retinal neovascularization. Yang and colleagues observed 20 eyes from 17 patients with high-risk PDR and showed that the combination of intravitreal bevacizumab and PRP led to the rapid clearance of VH, regression of retinal neovascularization, and visual improvements in the treatment of high-risk PDR^20^.

The DRCR Network developed Protocol S to compare intravitreal ranibizumab with PRP for the treatment of PDR in 394 eyes. At 2 years, the visual outcome of the ranibizumab-treated eyes was non-inferior to the PRP-treated eyes, with a mean visual acuity difference of +2.2 letters in favor of the ranibizumab group. The results of this trial suggest ranibizumab injections are a safe and effective alternative to PRP for the management of PDR^9^.

Conbercept is a soluble receptor decoy that blocks all isoforms of VEGF-A and VEGF-B as well as PlGF, which has a high binding affinity for VEGF and a long half-life in the vitreous body^21^. It was approved to treat neovascular AMD by the China State FDA in December 2013. Compared with aflibercept, conbercept is larger and contains the fourth binding domain of VEGFR-2. It has a lower VEGF dissociation rate, higher binding affinity, decreased adhesion to the extracellular matrix and lower isoelectric point, which results in a longer clearance time^22^. Relatively few studies have been performed on the intravitreal injection of conbercept for the treatment of neovascularization in PDR. Some studies have focused on preoperative conbercept pretreatments and found that it could be an effective adjunct to vitrectomy by reducing the chances of intraoperative bleeding, accelerating postoperative vitreous clear-up and helping stabilize visual acuity restoration for PDR^23,24^.

In our study, 15 PDR patients (15 eyes) with active NVD were treated with intravitreal conbercept injections (0.5 mg) followed by laser treatment at least 2 weeks after injection. All NVD regressed to different degrees, over a mean period of 7.5 days, manifesting as a significant decrease in the mean NVD area. This showed that conbercept can quickly cause regression of NVD. However, one eye required vitrectomy surgery during follow-up due to a dense, persistent vitreous hemorrhage and proliferative membrane formation. The recurrence of NVD was observed in two eyes due to insufficient laser photocoagulation, and NVD regressed after repeated conbercept injections. At the last visit, at a mean time of 8.3 months, all eyes had anatomic (determined by OCTA) improvements and visual acuity stabilization, demonstrating that the combination of intravitreal conbercept injections and laser photocoagulation is a safe and effective treatment for PDR. In addition, we found that the fibrous membranes on the disc were removed easily with little hemorrhaging during the surgery.

The recurrence of neovascularization is an important concern in anti-VEGF treatments in PDR. Arevalo *et al*.^19^ and Arevalo and Garcia-Amaris^7^ found that 21 eyes (47.7%) needed a second injection of bevacizumab, and 7 eyes (15.9%) needed a third injection due to the recurrence of neovascularization. In Yang’s study, recurrent retinal neovascularization with minor preretinal hemorrhaging was observed in 6 eyes (30%) 3 months after the first injection, which resolved after repeated bevacizumab injections^20^. In the current study, the NVD of 2 eyes (13.3%) recurred after the initial conbercept treatment and was stable after a second injection combined with laser photocoagulation. Kubota and associates demonstrated that vascular endothelial cells with decreased expression of VEGF were still present in the proliferative tissues after a bevacizumab injection. Anti-VEGF therapy temporally reduces the blood flow of the new vessels; however, it does not induce the complete regression of the vascular endothelial cells in new vessels^25^. We consider PRP to play an important role in preventing the recurrence of neovascularization.

Tractional retinal detachment after injection is another concern of anti-VEGF treatments. Theoretically, the anti-VEGF pharmacologic agent has fibrosis-inducing abilities and increases the probability of tractional retinal detachment. In the literature, tractional retinal detachment was reported in some PDR cases after intravitreal bevacizumab^19,20^. In the current study, one eye had proliferative membrane formation without obvious tractional retinal detachment after conbercept injection. We reason that preexisting fibrovascular tissue contractions and vitreoretinal adhesions may be risk factors in tractional retinal detachment, and attention should be drawn before anti-VEGF treatment in this kind of PDR patient.

As we decided to quantitatively evaluate changes in abnormal vessels in PDR after treatment, OCTA was chosen for the measurements of the area of neovascularization. The introduction of OCTA into the clinical practice is useful to monitor different NVD subtypes, their development, and the efficacy of treatment regimens as well as to define new vessel morphological details without the need for dye injections and to avoid dye leakage^12,26^. Using OCTA, the number, course, size and extension of NVD can be determined, but these features cannot be properly assessed using FA^12,26^. Because of the limits of the extent of OCTA scanning, not all neovascularizations could be quantitatively evaluated, so we chose NVD as the study metric. In our current study, the vascular structures of the NVD could be visualized clearly on OCT angiograms, even with a thin preretinal hemorrhage, and the area of the NVD could be calculated for statistical analysis. Another limit of OCTA is that it cannot show leakage of the neovascularizations, so it is not able to assess the activity of the NVD directly. In a recent study, Ishibazawa and colleagues have put forward the concept of exuberant vascular proliferation (EVP), which is the intense growth of irregular small-caliber vessels located at the margin of new vessels, likely representing active proliferation^13^. In our study, most of the patients did not have FA, so we were not able to compare OCTA results to the leakage in FA. However, EVP was present in 86.7% (13/15) of patients before treatment, and all these vessels converted to pruned vessels with residual, larger vascular loops approximately 1 week after conbercept injection, manifesting as the disappearance of EVP in OCTA^13^. While the concept of EVP is the assessment of the activity of the NVD qualitatively, we used NVD areas to compare changes in the NVD before and after treatment quantitatively. We noticed that all of the EVPs disappeared 1 week later, but NVD areas continued to decrease from 0.56 ± 0.17 mm^2^ to 0.22 ± 0.11 mm^2^ at last visit.

The limitations of this study include the fact that it had a relatively small number of patients and a short-term follow-up period. Meanwhile, there was no control group of NVD in PDR patients without intravitreal conbercept treatment. In addition, we hope that an automatic quantitative method to evaluate NVD and wide-angle OCTA to evaluate neovascularizations at different locations will become available in the future.

In conclusion, conbercept is a well-tolerated medication that causes a quick regression of NVD, and intravitreal conbercept injections combined with intensive PRP is an effective treatment for NVD in PDR patients. Quantitative information on NVD can be obtained with OCTA, which may be clinically useful in evaluating the therapeutic effect of treatments for NVD in PDR. A prospective study with long-term follow-up is needed to confirm the therapeutic benefits of conbercept and to evaluate ocular and systemic adverse effects.
